# State-of-the-art on analytic hierarchy process in the last 40 years: Literature review based on Latent Dirichlet Allocation topic modelling

**DOI:** 10.1371/journal.pone.0268777

**Published:** 2022-05-27

**Authors:** Peter Madzík, Lukáš Falát

**Affiliations:** 1 Department of Business Administration and Management, Technical University of Liberec, Liberec, Czech Republic; 2 Department of Macro and Microeconomy, University of Žilina, Žilina, Slovakia; Hefei University of Technology, CHINA

## Abstract

Although there are several articles that have carried out a systematic literature review of the analytical hierarchy process (AHP), many of them work with a limited number of analyzed documents. This article presents a computer-aided systematic literature review of articles related to AHP. The objectives are: (i) to identify AHP usage and research impact in different subject areas; (ii) to identify trends in the popularity of the AHP from the first introduction of the method in 1980 to the present; (iii) to identify the most common topics related to AHP and topic development over time. We process 35,430 documents related to AHP, published between 1980 and 2021, retrieved from the Scopus database. We provide detailed statistics about research interest, research impact in particular subject areas over the analyzed time period. We use Latent Dirichlet Allocation (LDA) using Gibbs sampling to perform topic modeling based on the corpus of abstracts. We identify nine topics related to AHP: Ecology & Ecosystems; Multi-criteria decision-making; Production and performance management; Sustainable development; Computer network, optimization and algorithms; Service quality; Fuzzy logic; Systematic evaluation; Risk assessment. We also present the individual topics trends over time and point out the possible future direction of AHP.

## 1 Introduction

The Analytic Hierarchy Process (AHP) method is currently one of the most frequently used decision support tools. Saaty [1] proposed the initial logic of the method, and three years later, the AHP method was generalized as a universal decision support tool. AHP is based on three principles that one can recognize in problem-solving—decomposition, comparative judgments, and synthesis of priorities [2]. The procedure proposed by Saaty significantly simplifies prioritization in multiple criteria decision-making. To date, many research papers have been published that have used this method and have focused on areas such as selection, evaluation, benefit-cost, allocation, planning and development, priority and ranking, decision making (general), forecasting, medicine, or quality function deployment [3]. The application of AHP is also not limited by industry and finds application in virtually any research area [4].

If we look at the use of the AHP method in research and practical applications, we can state that the usage of the AHP is very widespread. The most common articles to cover state-of-the-art in any field are systematic reviews. Systematic review studies or meta-analysis studies focused on AHP have in the past sought to capture the main currents and directions in the use of this method. Although many presented AHP applications and identified mainstream streams, most of them could have two main limitations. The first one could be questionable representativeness. Systematic literature review articles are most often analyzed articles in the most reputable journals. Although they captured the strongest trends, analysis of this type rarely covered more than 100 such articles. At the same time, it should be noted that the primary objective of systematic review is synthesizing evidence [5, 6]. Systematic reviews are often focused on answering specific issues in a specific field. General research questions are rarely the subject of systematic reviews. On the other hand, it is understandable that a systematic literature review, which would contain thousands of articles, would be extremely time-consuming in terms of implementation. The second limitation is the timeliness of the findings found in the systematic literature review or meta-analysis review articles. The longer it has been since the study was published, the less current its conclusions are. In connection with the intensive growth of the use of AHP, the need for up-to-date trend capture is becoming increasingly important. This article focuses on the data-driven comprehensive review of AHP use, analyzing huge amount of Scopus documents. Compared to standard systematic reviews, this study could provide a broader picture of AHP method.

### 1.1 Foundations of analytic hierarchy process

AHP has undergone dynamic development since its inception, but in the 1980s, researchers focused more on developing the principles and foundations of this method. At a certain degree of simplification, it can be stated today that the method has three basic principles and three axioms [7, 8]. The first principle is comparative judgments to determine the "local" priorities (weight) of the elements. The other two principles—the principle of hierarchical composition and the principle of synthesis—make it possible to process local priorities into "global" priorities. To apply these principles, researchers often refer to three axioms. The first is the reciprocal axiom, which requires a pairwise comparison of elements. The second is the homogeneity axiom. It should not be used to compare widely disparate elements [9]. The third is the synthesis of axioms that states that judgments about or the priorities of the elements in a hierarchy do not depend on lower level elements [9]. While the first two axioms are generally fully sufficient for practical purposes, according to Forman and Gass [8], the third axiom should evoke discourse.

One of the main advantages of AHP is its flexibility, logic, and ease of application, which has been reflected in the significant growth of publications that use this method [4]. Decision-making can be found in virtually any research area—for this reason, the application of AHP has been applied in areas such as engineering [10], computer science [11], business and management [12], mathematics [13] or social sciences [14]. The possibilities of AHP adjustments are also relatively wide, for example, through fuzzy logic [15], sensitivity analysis [16], or application to problems associated with risk assessment [17], or design [18]. However, these AHP applications represent only a selection of the most common and more detailed information on the possibilities of AHP is provided by systematic literature review papers on this method.

### 1.2 State of the art of AHP reviews

The development of the AHP method and its application had a relatively wide application in the 1980s. However, these applications relied heavily on developing the mathematical foundations of AHP [19]. The first review article on the possibilities of applying AHP was published by Vargas [20]. In his work, he summarized the methodological foundations of the use of AHP and its axioms and synthesized research articles published so far. The results pointed out that AHP can be used to solve economic/managerial, political, social, or technological problems [20].

The growing interest in AHP applications is documented by a brief look at the Scopus bibliographic and citation database. Between 1980 and 1990, 109 articles related to AHP were registered in this database. In the next period 1991–2000, the increase in such records was almost 6-fold (a total of 604 articles). This increase is mainly characterized by the extension of the AHP method to other scientific areas. Concerning the impact of the articles measured over the number of citations, some of the most important articles can be described in more detail.

Forman and Gass [8] published a study, the aim of which was to discuss why AHP is a general methodology for a wide variety of decisions and other applications, to present brief descriptions of successful applications of the AHP and to elaborate on academic discourses relevant to the efficacy and applicability of the AHP vis-a-vis competing methodologies. Based on the analysis of the successful use of AHP in various companies and institutions, the authors defined eight application areas: choice, prioritization/evaluation, resource allocation, benchmarking, quality management, public policy, health care, strategic planning. This practical part was extended by a scientific discourse focused on six areas: transitivity and rank reversal, transitivity, adding irrelevant alternatives and rank reversal, measurement and ratio-scales, prioritizing objectives/criteria, AHP with feedback (ANP) and approximations. The study’s strength is a relatively detailed overview of the principles and foundations of AHP and an attempt to define the application areas.

Another review study was published by Vaidya and Kumar [3]. It aimed to present a literature review of AHP applications. The authors analyzed 150 selected articles related to AHP, which were published before 2003. The articles were subsequently analyzed according to three aspects: applications based on a theme; specific applications; applications combined with some other methodology. The presented results were divided into ten application areas: selection; evaluation; benefit-cost analysis; allocations; planning and development; priority and ranking; decision-making; forecasting; medicine and related fields; AHP in QFD applications. According to this categorization, it can be seen that the views on the classification are partially mixed and include a purpose perspective (selection, evaluation, allocation, etc.) as well as a sectoral or sectoral perspective (medicine, QFD). The study also contains an overview of the most frequently used journals for publishing topics related to AHP, which is positive. The authors also tried to outline the development of the topic of AHP over time but used only a simple overview in the form of a pie chart, which covers periods of three to four years. On the other hand, the authors should be commended for the content analysis of a large number of articles, without which the definition of thematic groups would not be possible.

In 2010, the Turkish authors Sipahi and Timor [21] published another review study on the current possibilities of using AHP and its extended version of ANP. This literature review included an analysis of 232 application articles related to AHP or ANP in the period 2005–2009. Based on the content analysis of selected articles, the authors found that an exponential increase in the application of AHP can be observed in the observed period. The article offers a relatively good overview of the original sources in each area, and the structure is somewhat reminiscent of the study by Forman and Gass [8]. The authors supplement the results with the combination possibilities of AHP, as they also give examples when this method is used together with other tools such as simulation, TOPSIS, GIS, Goal programming, etc. The positive aspect of this study can be considered the relatively high number of analyzed articles. On the other hand, the negative of this literature review can be considered a narrow period of time, which can offer the current state of the AHP application, but without the possibility to capture the past development of this topic.

While previous literature analyzes have focused on defining the application areas of AHP, the study by Ishizaka and Labib [16] focused more on methodological developments of AHP. The study aimed to conduct a neutral review of nine methodological topics that the researchers had addressed in the past. These topics include problem modeling, pairwise comparison, judgment scales, derivation methods, consistency indices, incomplete matrix, synthesis of the weights, sensitivity analysis, and group decisions. Although the authors deal with these topics mathematically, they also state that the success of the use of AHP is its simplicity, hierarchical modeling of the problem, and the possibility of adopting verbal judgments. This review study offered a relatively new and original overview of the use of AHP not through a purposeful and sectoral perspective but through methodological issues.

One of the broadest review studies on the AHP applications includes a literature review with a social networks analysis, published by Emrouznejad and Marra [4]. This study aimed to trace the pattern of development of AHP research, identify the patterns of collaboration among authors, identify the most important papers underpinning the development of AHP and discover recent areas of interest. Regarding the number of articles examined, this study is the most extensive of all mentioned—8441 papers published between 1979 and 2017 retrieved from the ISI Web of Science database were analyzed. The results, to some extent, confirmed previously published findings regarding the development of the AHP topic. The authors identified in the first time period (1979–1990) that attention was focused on the development of the theoretical foundations of AHP. In the second period (1991–2001), there was an increase in the application of AHP in areas such as computer science, mathematics, business, and management studies and its introduction in new research areas. The third period, which covered the years 2002–2017, was characterized by expanding AHP into areas such as fuzzy logic, TOPSIS, DEAHP, SWOT, QFD, sensitivity analysis.

Five studies were presented above, which focused on a systematic analysis of the development of topics related to AHP. These studies were generically focused on the comprehensive capture of AHP without deeper and more detailed specialization. For the sake of completeness, however, it should be noted that the topic of AHP and its applications was also analyzed from a more detailed perspective, either from the perspective of a specific subject area or other characteristics. Below is a selection of some overview articles with a more specific focus:

- Apostolou and Hassell [22] summarized the use of AHP in accounting research through a chronological arrangement
- Ho [13] focused on the analysis of articles in which AHP is combined with other tools such as QFD, DEA, or SWOT
- The classification of healthcare articles according to several classification criteria (publication year, journal, method of analyzing alternatives, etc.) was published in their study by Liberatore and Nydick [23]
- Subramanian and Ramanathan [10] analyzed the development of articles in operations management and pointed to the trend of using AHP when problems require considerations of both quantitative and qualitative factors.

### 1.3 Research gap

From the review studies described above, one can see an effort to cover the topic of AHP as widely as possible. As the systematic literature review studies that analyze the AHP application usually included only a few dozen studies, the representativeness of the results may not always be guaranteed. Most authors of such review studies seek to address this shortcoming by including studies in the most reputable journals in the analysis. As these are highly renowned journals, this may partially reduce the representativeness problem, but it will not completely eliminate it. One of the few studies that have eliminated such a deficiency is a review conducted by Emrouznejad and Marra [4]. However, the authors of this study apparently had to proceed with simplification for interpretation reasons and divided the results into three groups according to the time period. Although the results are more complex, it was difficult to capture trends in the development of AHP.

However, bibliographic and citation databases currently offer much broader analytical possibilities for processing scientific trends in various topics or areas. Given the enormous growth of articles published on the topic of AHP over the last five years, the need to capture the trends in the application of AHP with regard to its past development is extremely topical. Our study reflects the need for a review of AHP—we use a big-data approach to go beyond the scope of systematic reviews. A data-driven machine learning approach was used to get a broader picture of AHP usage. In this article, we focus on three areas (research questions) that have so far been insufficiently taken into account in the comprehensive analysis of AHP:

- RQ1: What is the usage of AHP and research impact in individual subject areas?
- RQ2: What are the trends in AHP popularity from the first introduction of the method in 1980 to the present?
- RQ3: What are the most common topics related to AHP, and what is their development over time?

Focusing on these three research questions will make it possible to update previous results and broaden the context of AHP applications by examining a many more articles. Therefore, it can be assumed that the results will show a higher degree of representativeness than the review articles published so far.

## 2 Methodology

**Fig 1:**
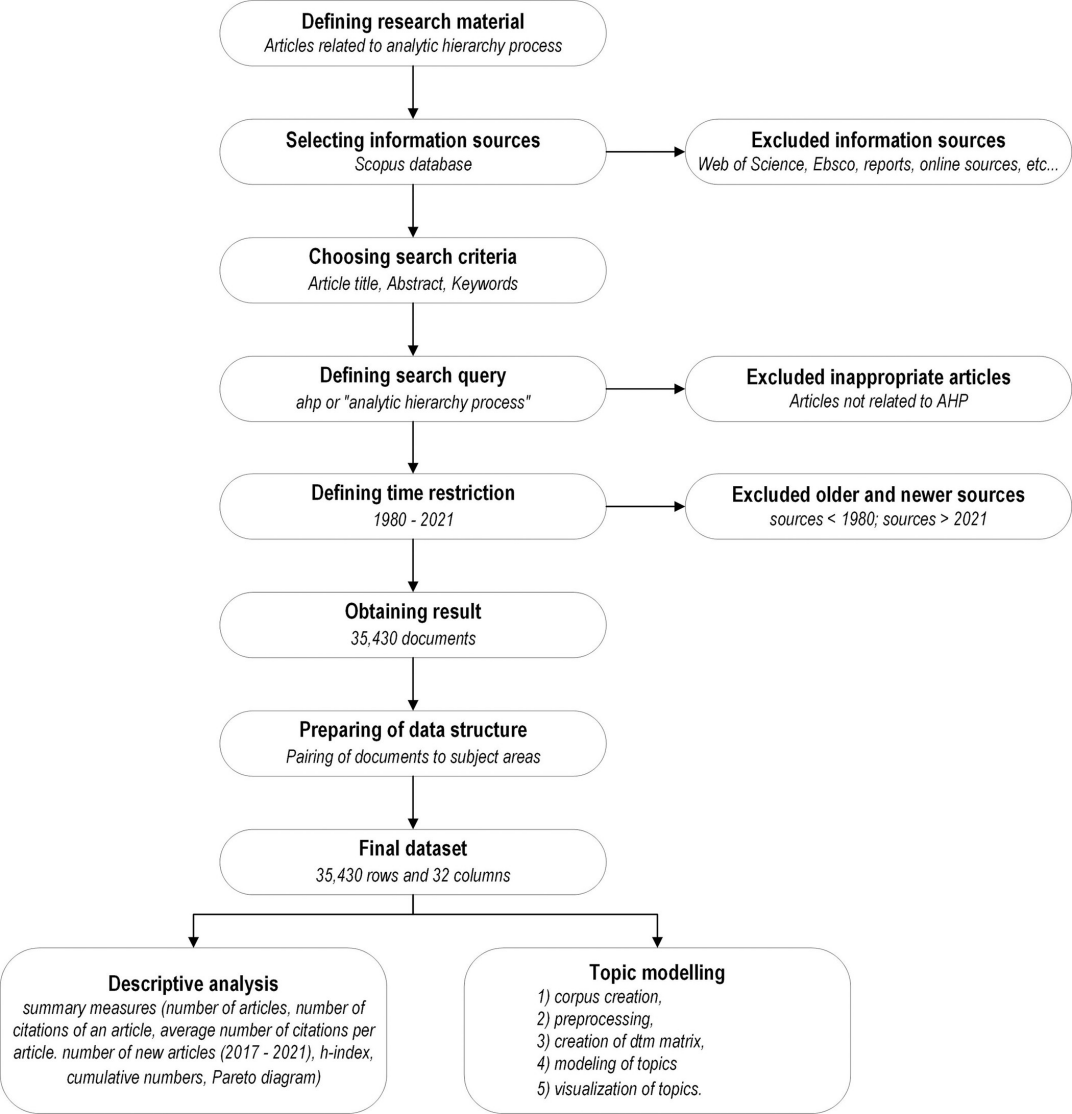
Research design protocol.

To cover the defined research questions, a procedure consisting of three main phases was determined—data acquisition, variables (dataset structure), and data analysis. These three phases are described in more detail in Chapters 2.1 to 2.3. Particular methodology steps are shown in Fig 1.

### 2.1 Data acquisition

The acquisition process data consisted of two steps, with the data downloaded from the Scopus database. Scopus is one of the most prestigious and largest scientific databases and contains information on abstracts and citations and other metadata on scientific articles. Currently, this database includes more than 76 million records. Indexing sources come from more than 39,100 journals, 120,000 conferences, and 206,000 books [24].

As a first step, we focused our search on documents of scientific articles published between 1980 and 2021. The data presented in this article were collected on October 12, 2021. Data retrieval strategy in the Scopus database was as follows. After setting up a document search, we set the search criteria to Article title, Abstract, Keywords. We then defined a search query: ahp or "analytic hierarchy process". The returned results were further modified by removing documents that were published before 1980 (the year of the first publication of the AHP). We have obtained 35,453 documents. Finally, documents that had the release year of 2022 were removed. The resulting dataset was 35,430 documents in size. Our selection process was not limited due to the type of studies, i. e. the suitability of the studies for our sample was not limited to systematic reviews or meta-analyses.

In the second step, we obtained a database of resources indexed in Scopus. The data in this database contained the name of the source and its assignment to one or more of the 26 subject areas.

### 2.2 Variables

The final dataset was created by merging the two datasets described above. Data on subject areas have been paired to the document records dataset. The dataset contained 35,430 rows and 32 columns. The rows represent the documents, and the columns attributes of the individual documents. Attributes (variables) defined the basic information about the article, i. e.: authors, title, year, source title (journal name), the number of citations, the text of the abstract, and 26 subject areas, which were defined as follows: Agricultural and Biological Sciences (AGRI); Arts and Humanities (ARTS); Biochemistry, Genetics and Molecular Biology (BIOC); Business, Management and Accounting (BUSI); Chemical Engineering (CENG); Chemistry(CHEM); Computer Science (COMP); Decision Sciences (DECI); Dentistry (DENT); Earth and Planetary Sciences (EART); Economics, Econometrics and Finance (ECON); Energy (ENER); Engineering (ENGI); Environmental Science (ENVI); Health Professions (HEAL); Immunology and Microbiology (IMMU); Materials Science (MATE); Mathematics (MATH); Medicine (MEDI); Neuroscience (NEUR); Nursing (NURS); Pharmacology, Toxicology and Pharmaceutics (PHAR); Physics and Astronomy (PHYS); Psychology (PSYC); Social Sciences (SOCI); Veterinary (VETE). If the publication was included in the selected subject area by the Scopus database, this was marked for the given document. It is important to recall that one article could be included in more than one subject area.

### 2.3 Data analysis

Data analysis was performed in two phases. The first phase involved descriptive and exploratory data analysis. The main summary measures were the following three main metrics: number of articles, number of citations of an article, average number of citations per article. In addition, we used the metrics number of new articles (2017–2021) and the h-index [25].

In addition to the main summary measures, we used cumulative numbers and a Pareto diagram to synthesize the results of the descriptive analysis. Next, we structured the results of the descriptive analysis according to subject areas and individual years. When structuring by subject areas, we monitored the number of articles in the subject area, the average number of citations per article in the subject area, the number of new articles (2017–2021) in the subject area, the Hirsch index in the subject area. We also used relative statistics in the structured analysis according to subject areas (cumulative percentage of articles by subject area, share of articles in selected subject area over total articles, share of citations in selected subject area over total citations). When structuring by individual years, we monitored the development of the total number of articles published in selected subject areas for individual years. Finally, we analyzed the journals with the highest impact on AHP dissemination based on the total number of article citations.

The second phase of data analysis was topic modeling using the Latent Dirichlet Allocation (LDA) method. It is an unsupervised machine learning method of probabilistic clustering and is a type of Bayesian model. The principle of the method is that each element of the dtm (document-term matrix) matrix is a mixture of a finite number of topics with a certain probability. Each topic is a mixture of several words with a certain division. [26]

LDA was defined by Blei, Ng and Jordan [26] and is characterized as follows: Let *θ* be a random variable that has a k-dimensional Dirichlet probability distribution. This variable then has the following probability density on the simplex [26]:

$$p(\theta |\alpha )=\frac{\Gamma (\sum_{i=1}^{k}\alpha_{i})}{\prod_{i=1}^{k}\Gamma (\alpha_{i})}\theta_{1}^{\alpha_{1}-1}\ldots \theta_{k}^{\alpha_{k}-1}$$ (1)

where the alpha parameter is a *k*-dimensional vector.

If we define the basic parameters of the topic corpus, *alpha* (document-topic density) and *beta* (topic-word density), then the continuous distribution of the probability mixture of points *θ*, the set *N* of topics *z*, and the set *N* words *w* can be calculated as follows

$$p(\theta ,z,w|\alpha ,\beta )=p(\theta |\alpha )\overset{N}{\underset{n=1}{\prod }}p(z_{n}|\theta )p(w_{n}|z_{n},\beta )$$ (2)

Marginal distribution of a document is then defined as

$$p(w|\alpha ,\beta )=\int p(\theta |\alpha )(\overset{N}{\underset{n=1}{\prod }}\underset{z_{n}}{\sum }p(z_{n}|\theta )p(w_{n}|z_{n},\beta ))d\theta$$ (3)

The probability of the whole corpus is then defined as

$$p(D|\alpha ,\beta )=\overset{M}{\underset{d=1}{\prod }}\int p(\theta_{d}|\alpha )(\overset{N}{\underset{n=1}{\prod }}\underset{z_{n}}{\sum }p(z_{n}|\theta_{d})p(w_{n}|z_{n},\beta ))d\theta_{d}$$ (4)

where *D* is the corpus and *d* = 1… *M* are the individual documents of the corpus.

The practical implementation of extracting topics from data was performed in five steps: corpus creation, preprocessing, creation of dtm matrix, modeling of topics, and visualization of topics. All the above procedures were implemented in R language. For corpus creation and basic data preprocessing we used the tm package, which is a textmining package [27]. We used the SnowballC package to implement stemming, and the topicmodels package to model the topics themselves. We used LDAvis, servr, dplyr, strings, magrittr packages for visualization.

The first step that preceded the modeling of topics was the creation of a corpus, which is the set of all documents (abstracts). In our case, the corpus contained 35,430 documents and consisted of a set of all abstracts that were the input for our analysis.

The second step was to pre-process the data in the corpus, as the text data is unstructured data and, in essence, contains several problems for computer processing. In the preprocessing phase, all words in the whole corpus were transformed into lowercase, special characters (-,:, ‘,” -”, ©) were removed, punctuation was removed, numbers were removed, and additional spaces were removed. Subsequently, stemming was performed, in which the words were truncated to the word base. Subsequently, the differences between American and Australian English were removed, and non-meaningful words (stopwords) were removed from the corpus. In addition to the standard stopwords from the tm package, we also defined our own stopwords that have been removed from the corpus. Furthermore, we removed a set of so-called ahp stopwords because these words did not explain the topics, as they were found in every article.

The third step was to create a document-term matrix (dtm), whose rows contained documents (abstracts), and the columns formed words from the corpus. For computational efficiency, we decided to limit in dtm the maximum document frequency of a word to the number of documents (35430) and the minimum document frequency of a word to 1 percent (i.e. 35) of documents. We’ve also limited the minimum word length to 4 and the maximum word length to 20 characters.

The fourth step was the modeling of topics using the LDA method. The LDA requires defining *k* number of topics before running the method. Our goal was to choose such *k* which allow identified specific and understandable topics. At the same time, however, care must be taken to ensure that the number of topics is not too high due to incomprehensibility and complicated interpretation of the results. We decided to choose *k* from the set {8, 9, 10, 11, 12}. We used the Gibbs sampling method to quantify the parameters of the LDA method. "Gibbs sampling is a simulation tool for obtaining samples from a nonnormalized joint density function." [28]. The Gibbs sampling process in the LDA model can be expressed as

$$p(z_{i}=K|w,z_{-i})\propto \frac{n_{-i,K}^{\left(j\right)}+\delta }{n_{-i,K}^{\left(.\right)}+V\delta }\frac{n_{-i,K}^{\left(d_{i}\right)}+\alpha }{n_{-i,.}^{\left(d_{i}\right)}+k\alpha }$$ (5)

[27, 29, 30]. *p(z|w)* is posterior distribution, *z*_*-i*_ is “the vector of current topic memberships of all words without the *i*^*th*^ word *w*_*i*_. The index *j* indicates that *w*_*i*_ is equal to the *j*^*th*^ term in the vocabulary. $n_{-i,K}^{\left(j\right)}$ gives how often the *j*^*th*^ term of the vocabulary is currently assigned to topic *K* without the *i*^*th*^ word. The dot implies that summation over this index is performed. *d*_*i*_ indicates the document in the corpus to which word *w*_*i*_ belongs.” [27].

Since Gibbs sampling starts at a random point, we decided to burn the first 100 steps of this process (these results did not well represent the properties of our probability distribution). Subsequently, we performed 2000 iterations of this procedure, and due to the correlation between the samples, we took only every 40th iteration for further use. We performed experiments with a number of topics from 8 to 12. In order to minimize the chance of getting stuck in the local minimum, we performed 5 runs for each value k, and we saved only the best result. For the replicability of the results, we defined the initial settings (seed) of the 5 run runs: 2003, 5, 63, 100001, 765. With regard to assessing the degree of cluster distinguishability based on the composition of the most frequent words in individual topics, we decided on the final number of topics.

The fifth step was to visualize the themes. Topics were visualized on intertopic 2D distance maps via multidimensional scaling using principal component analysis (PCA) via the LDAvis library. In the intertopic map, each topic was represented by top-30 most salient terms, where saliency was defined according to Chuang, Manning and Heer [31].

The final product of the quantified LDA method was a list of the abstracts of individual abstracts to the topic with the highest probability, a list of the most representative words to the given topic and a list of probabilities of the affiliation of each document to each topic.

## 3 Results

### 3.1 AHP research in subject areas and citation overview

**Fig 2:**
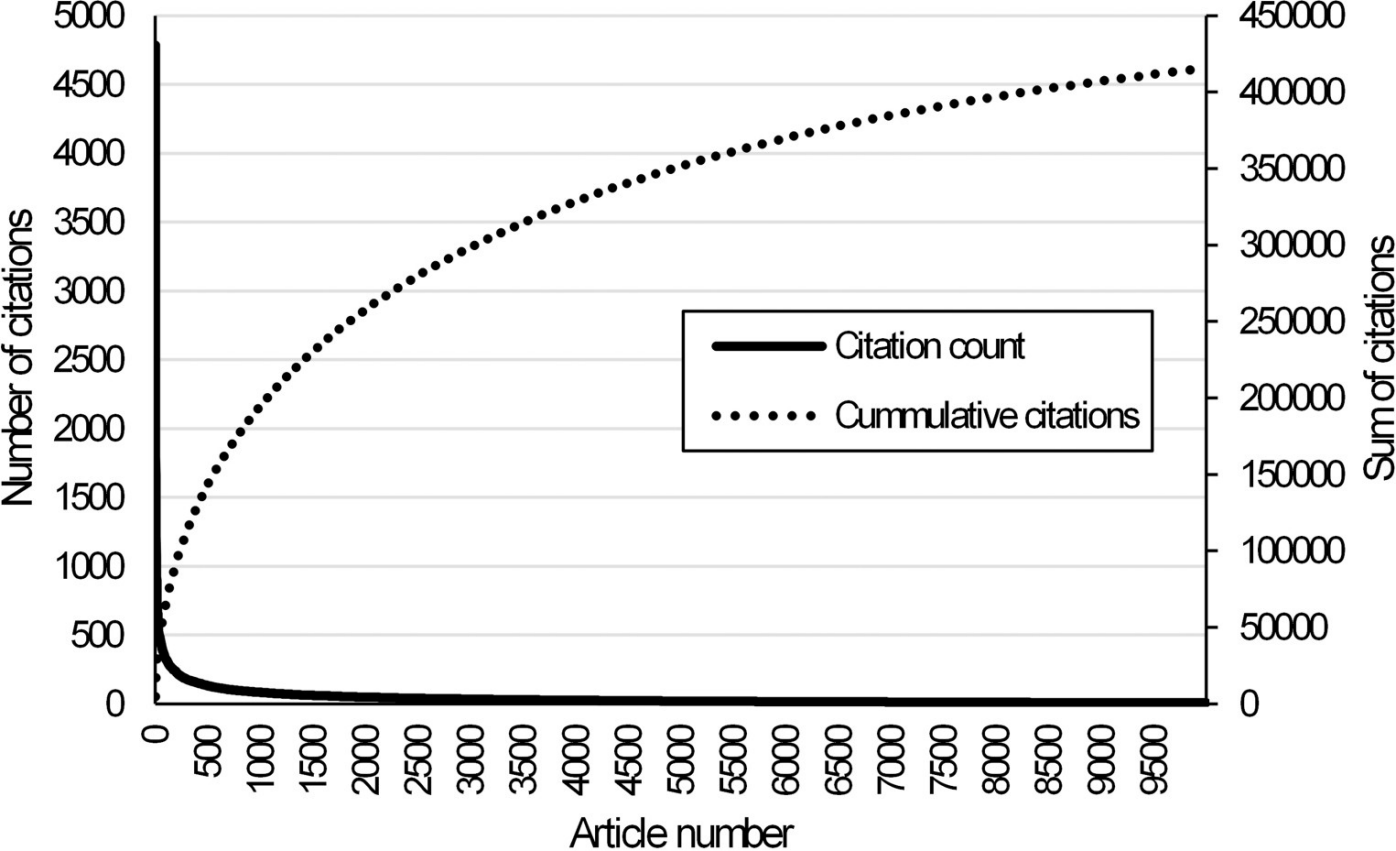
Articles vs. citations—Pareto overview.

35,430 records were included in the analysis, which contained the terms AHP or "Analytic hierarchy process". The number of records was current as of October 12, 2021, and these articles covered the period from 1980 to 2021. A total of 457,815 citations were registered for all these articles. Fig 2 shows the Pareto article distribution report (only the 10,000 most cited records were displayed). The first 5,784 most cited articles (16.3%) had a total of 80% of all citations (366,260 citations).

**Fig 3:**
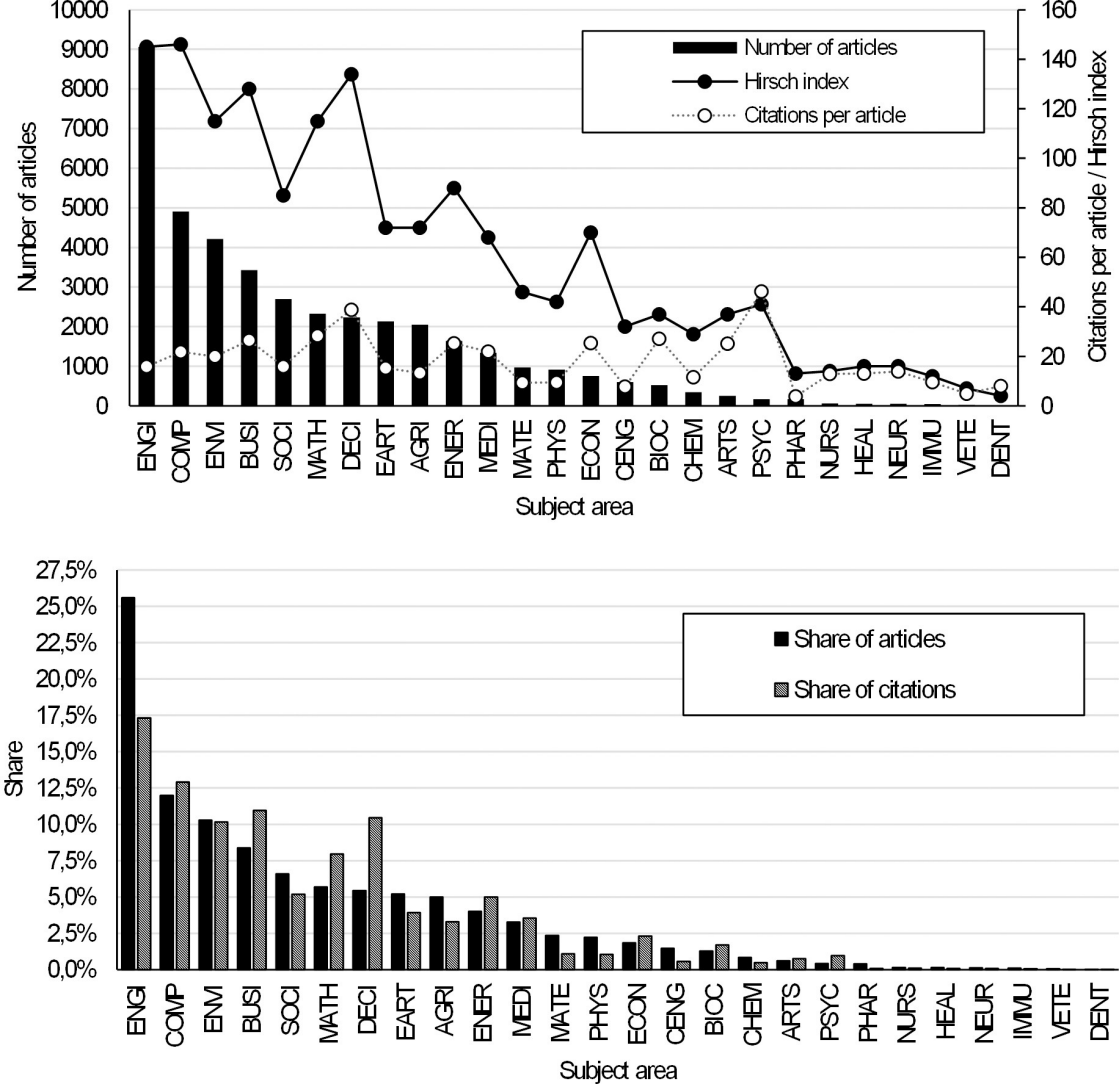
Subject areas and their share of all AHP articles.

Topics related to AHP have been included in articles in virtually all subject areas. The number of these areas was 26. Fig 3 provides an overview of the number of articles, their citations, and the Hirsch index (Hirsch 2005) for each subject area. The attractiveness of the AHP can be measured by the number of citations of articles from the subject areas. However, the Citation per article indicator can be distorted, especially in cases where the total number of articles is low or there are relatively few articles with a very high number of citations. From the point of view of the attractiveness of the AHP, the Hirsch index, which combines productivity and research impact, is a better indicator. At the top of Fig 3, we can see that AHP related themes appear most in the articles from the following subject areas: ENGI, COMP, ENVI, BUSI, SOCI, MATH, DECI and EART. The differences between the number of articles and their citations can be significant, as shown at the bottom of Fig 3. If we wanted to define the dominant research areas related to the topic of AHP, we could select those for which the Hirsch index is higher than e.g. 100 (i.e. at least 100 articles have at least 100 citations). According to such an approach, we could include ENGI, COMP, ENVI, BUSI, MATH and DECI, among the dominant areas.

AHP is a multiple-criteria decision-making tool, which finds application mainly in those areas in which efforts to objectify decision-making can be observed. Interestingly, however, AHP was originally most closely associated with MATH and DECI research areas. These research areas still account for a significant share of the total number of articles and the total number of citations. But we can see that AHP is a multidisciplinary topic. Research already covers areas such as ENGI, COMP, ENVI, and BUSI, which are not at all negligible in terms of academic performance and impact.

To better understand the use of AHP in various scientific fields, we can analyze the number of articles and the number of citations by the source in which they were published. Table 1 provides an overview of journals sorted by the number of citations.

**Table 1:** Journals with the highest impact on AHP dissemination.

Journal	Subject Area	Citations of articles	Articles	Top article	Citations of top article
European Journal of Operational Research	DECI, MATH	28189	220	[19]	4785
Expert Systems with Applications	COMP, ENGI	19943	256	[16]	626
Journal of Cleaner Production	BUSI, ENER, ENGI, ENVI	9828	239	[32]	922
International Journal of Production Economics	BUSI, DECI, ECON, ENGI	8490	78	[33]	885
Diabetes Research and Clinical Practice	BIOC, MEDI	7129	3	[34]	2746
International Journal of Production Research	BUSI, DECI, ENGI	6743	114	[35]	448
Renewable and Sustainable Energy Reviews	ENER	6528	68	[36]	1259
Computers and Industrial Engineering	COMP, ENGI	4486	95	[37]	210
Sustainability (Switzerland)	ENER	4398	529	[38]	158
Energy	ENER, ENVI	4346	110	[39]	301
Journal of Environmental Management	ENVI, MEDI	4328	92	[40]	367
Applied Soft Computing Journal	COMP	4269	69	[41]	471
Omega	BUSI, DECI	4184	31	[42]	871
International Journal of Advanced Manufacturing Technology	COMP, ENGI	4085	113	[43]	322

One anomaly can be observed from the table—namely, the journal Diabetes Research and Clinical Practice. Only three articles on AHP have been published there, but they have an enormous citation rate. If we exclude this extreme, the top-5 journals publishing topics on AHP include European Journal of Operational Research, Expert Systems with Applications, Journal of Cleaner Production, International Journal of Production Economics and International Journal of Production Research. These journals are among the top in their field of science, which only testifies to the relevance of AHP’s research potential.

### 3.2 Trends in AHP popularity

**Fig 4:**
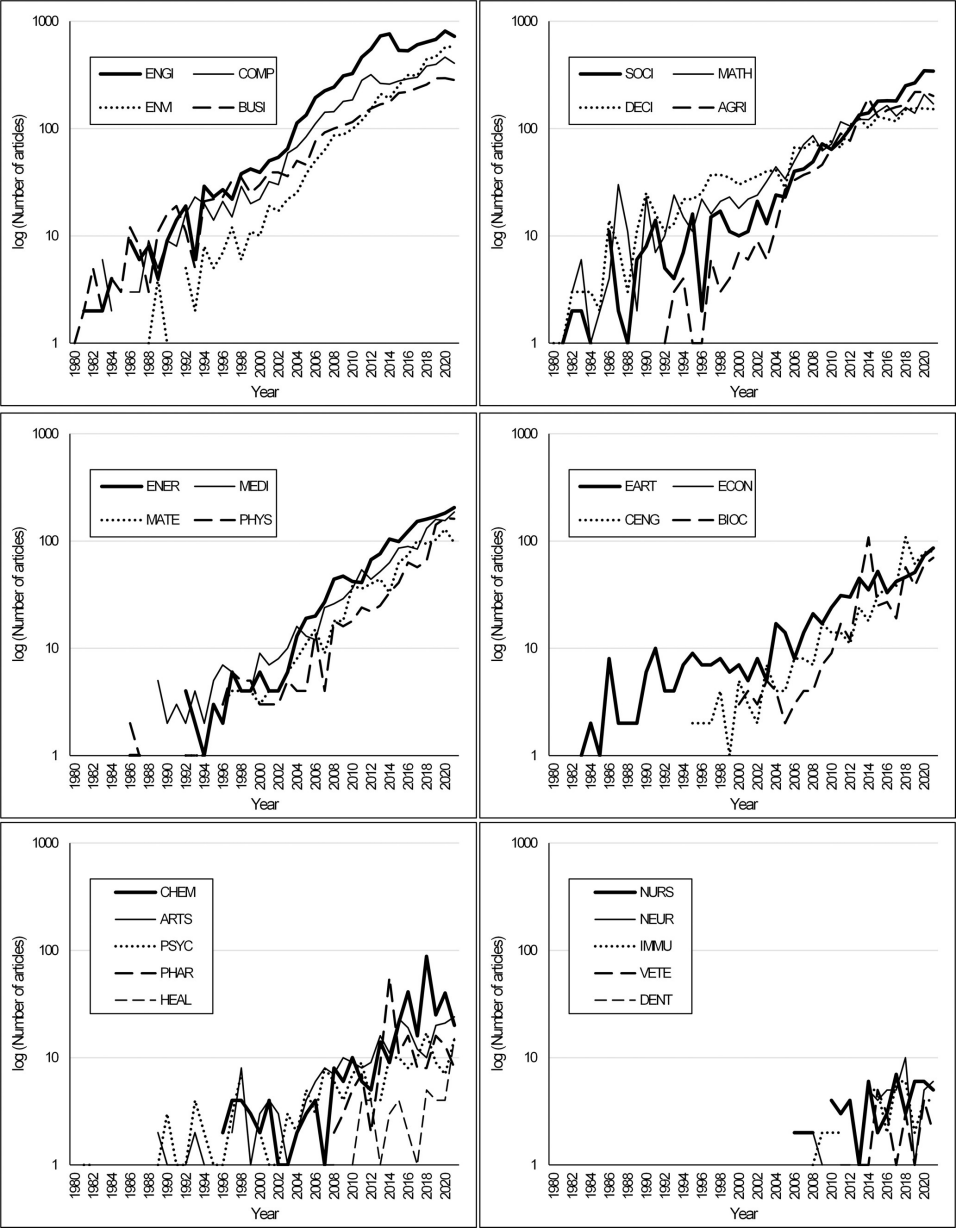
Number of articles development by subject areas.

To capture the use of the AHP method in the monitored 26 subject areas, we analyzed the number of articles in the given years. Fig 4 provides an overview of the development of all subject areas. The number of articles devoted to AHP is constantly growing in virtually all areas. We will discuss only a few of the most significant findings. In the area of ENGI, a deviation from the growing trend can be observed from 2015 to 2019. According to the development, it seems that the number of articles focused on AHP in this research area is currently approximately the same as in 2013 and 2014. Strong growth of interest in AHP can be identified in the area of ENVI and SOCI. Both areas have seen a significant increase in published articles since 2015.

The trend of publishing scientific articles has been growing for a long time. The Scopus database contains a total of 6.3 million records in 2020—articles, conference papers, reviews, editorials, notes, letters, etc.—in all subject areas. Twenty years ago—in 2000—there were only 2.1 million records and in 1980 only 0.9 million. The above results, therefore, need to be taken into account in view of this increase.

**Fig 5:**
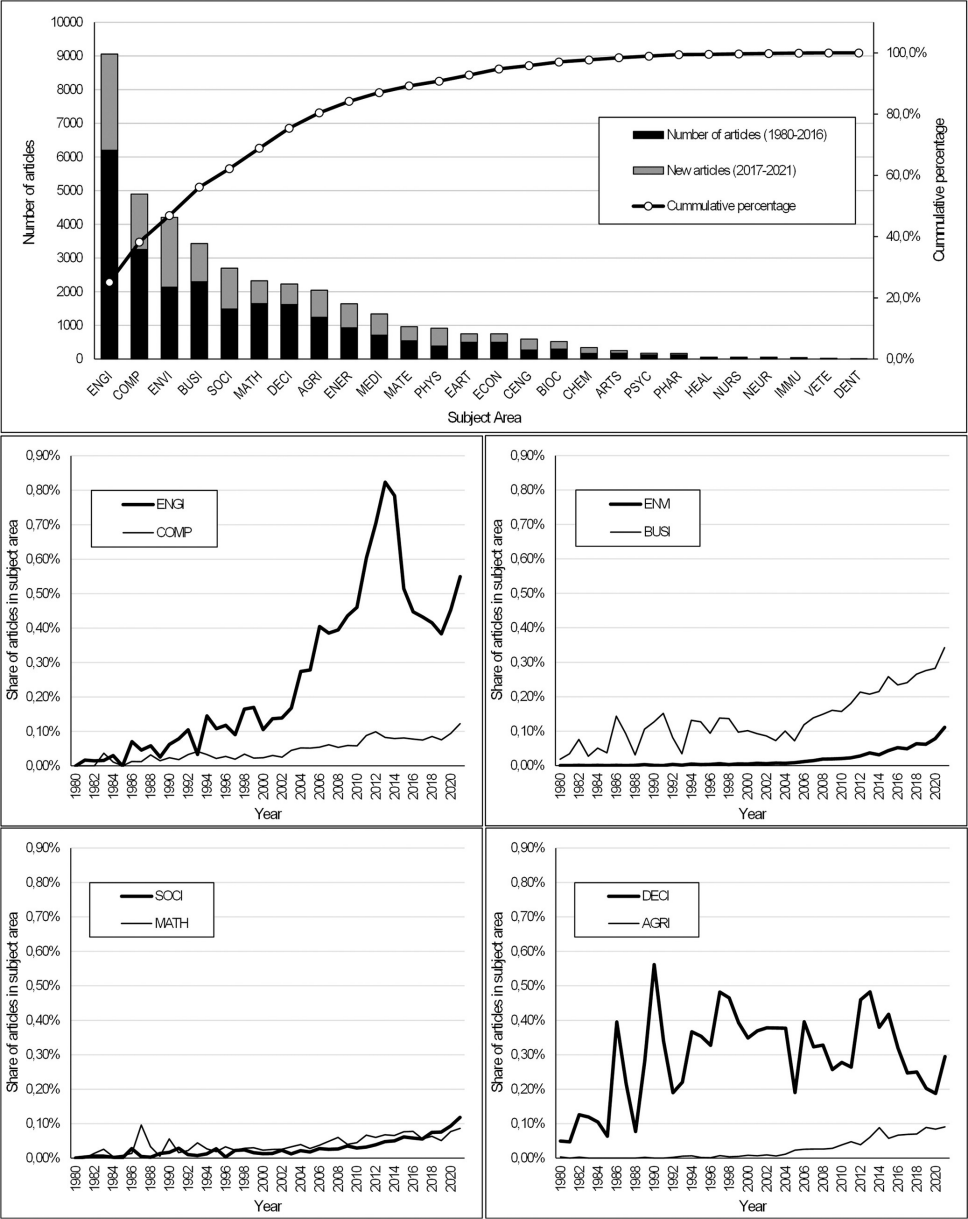
Development of the relative share of AHP in individual subject areas—absolute numbers for all subject areas (top); 8 most numerous subject areas (bottom).

Interest in AHP topics can also be analyzed by comparing the total number of articles in a given subject area in a given year and articles focused on AHP in a given year. Such a comparison will partially eliminate interpretation problems and help identify a real interest in the topics. The results are shown in Fig 5.

The topic of AHP is most used in ENGI (approx. 0.50% of all articles), BUSI (approx. 0.30%), and DECI (approx. 0.30%). ENGI had the greatest research interest in AHP in 2013 and 2014 (approximately 0.80%), while it subsequently fell sharply. The steady growth of research interest can be seen in the BUSI area, growing almost continuously since about 2005. It is also interesting to note that in the area of DECI, research interest in AHP has been hovering around the level of 0.30% for almost 40 years. This recalculation has not confirmed the increase in absolute article numbers previously identified in the ENVI and SOCI areas. There are currently a lot of published articles in the areas of ENVI and SOCI, with only a fraction directly or indirectly related to AHP (approximately 0.10%).

### 3.3 Topics related to AHP and their development

**Fig 6:**
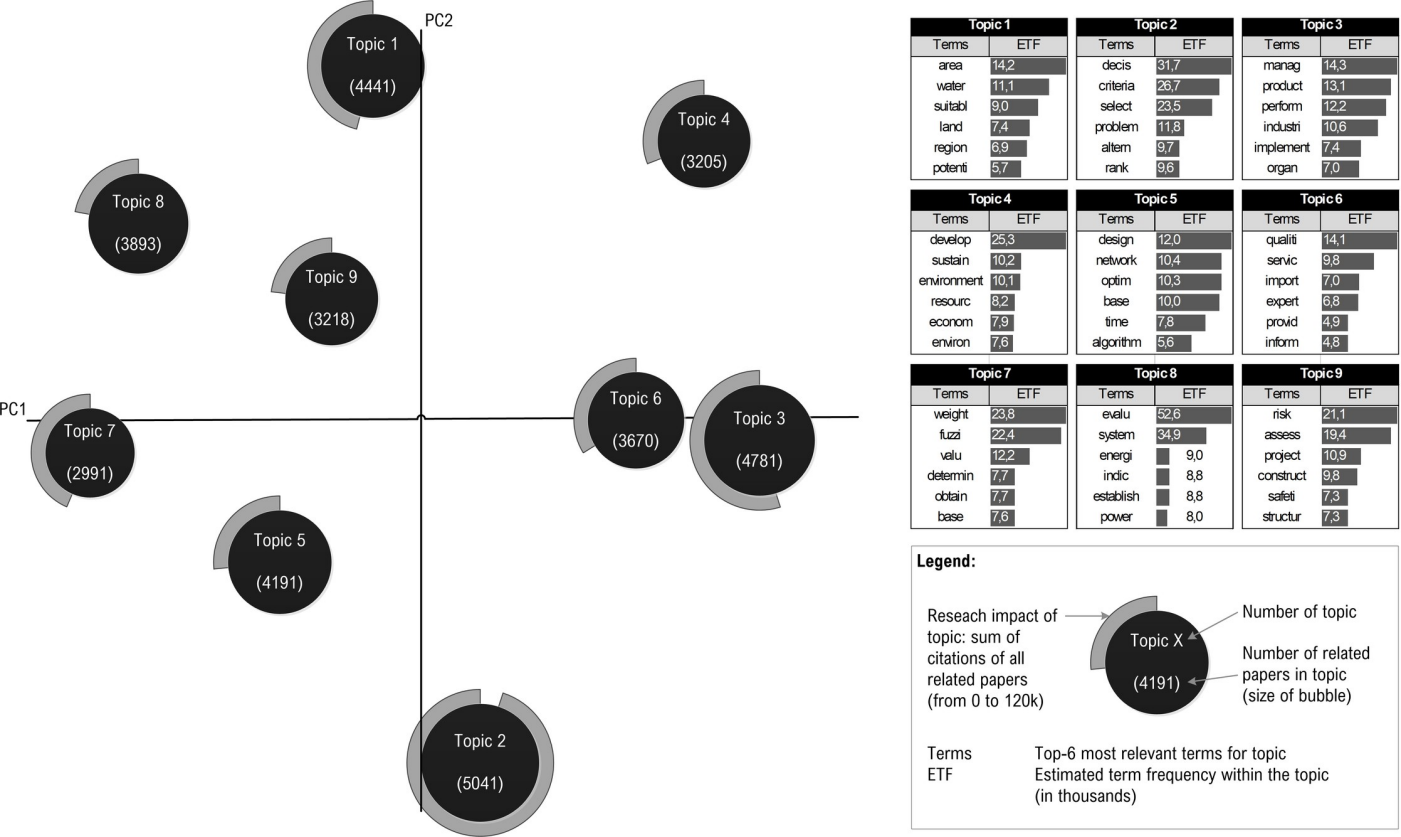
Intertopic distance map.

We used LDA topic modeling to analyze topics. Topic modeling is the process of identifying topics in a set of documents. Our set of documents consisted of 35,430 articles, and the LDA was used for abstracts of these articles. Several experiments have been performed with LDA to achieve a reasonable number of clusters with good interpretability and distinguishability. Based on the settings listed in section 2.3, we identified nine topics. Different terms with different frequencies characterized each topic. Fig 6 shows the display of topics via intertopic distance maps.

**Fig 7:**
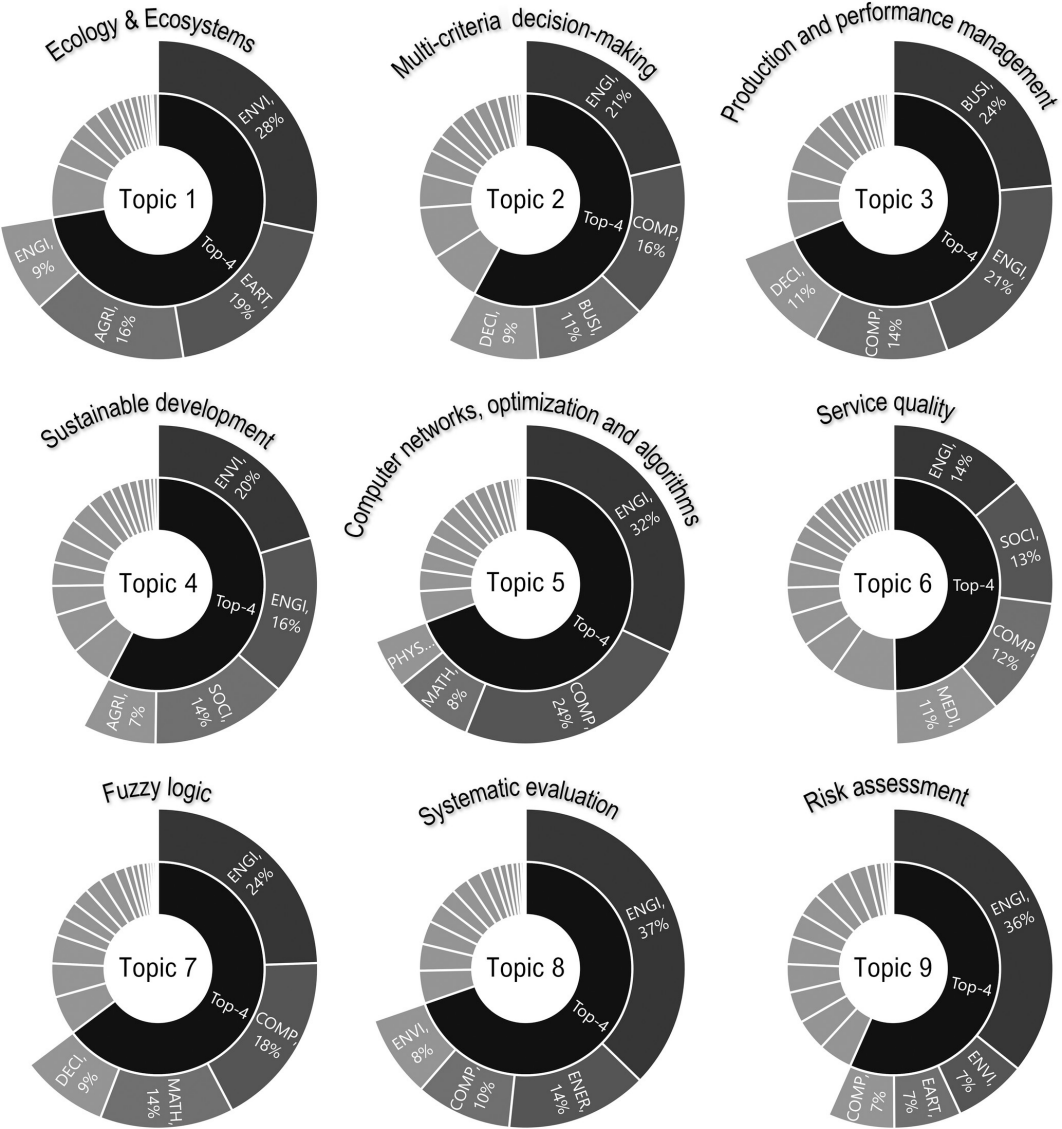
Names of topics and their composition of subject areas.

The words that were most frequent in a particular topic formed the basis for naming the topic. The higher the number of specific terms in the topic, the more we considered this term when naming the topic. Each article was assigned a probability of belonging to a given topic by the LDA algorithm. The article was assigned to the topic for which the probability was the highest. Based on this, it was possible to display the size of the topic (number of documents) and their research impact (number of citations). The identified topics were relatively independent, as the correlation coefficients between them reached low values—in the interval <-0.31; 0.06>. With regard to the most frequent words, the topics were named, and their representation in individual subject areas was assessed, while the top 4 subject areas were highlighted—Fig 7.

**Fig 8:**
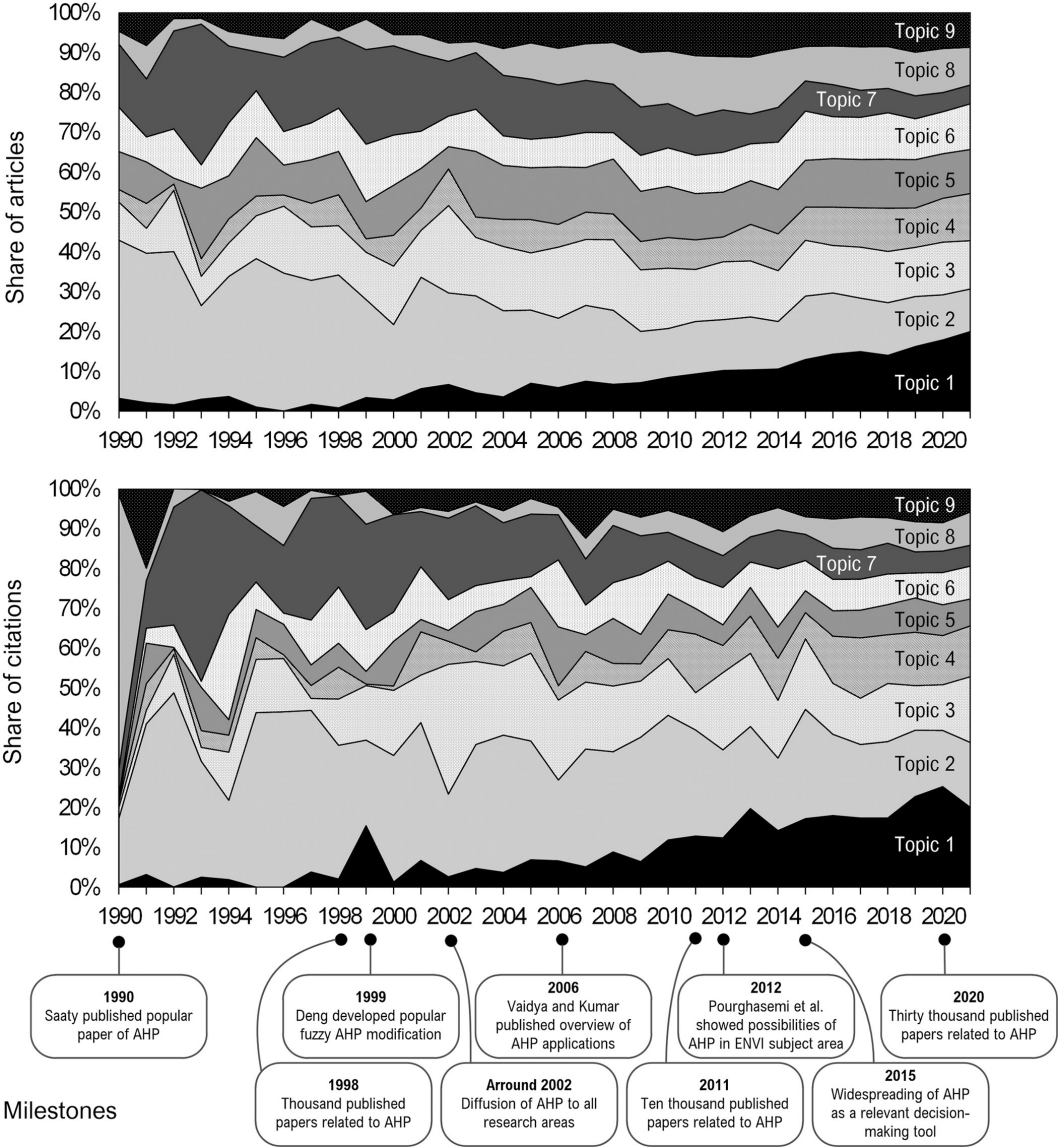
Topic interest (top) and topic impact (bottom) development.

Before we describe the topics and their characteristics, we looked at the development of topics over time. We analyzed the development from two perspectives—research interest and research impact. We analyzed the research interest through the relative number of articles on each of the nine topics in each year under review. The higher number (and proportion) of articles in a given year indicates a higher research interest in a given topic. We analyzed the research impact by the relative number of citations for all articles in a given topic and year compared to all citations for the whole year. The higher the number of citations in a topic, the higher the research impact of that topic. Fig 8 shows share charts representing the period from 1990 to 2021 (the period before 1990 had a relatively small number of articles, and the graphic results could therefore optically distort longer-term trends).

The Fig 8 shows the development of individual topics concerning their research interest and research impact. If we take a closer look, we can identify three types of topics: rising, stable, and declining.

We have identified two topics that have a rising character. These are Topic-01 and Topic-04, for which it can be stated that in the long run, their share is growing significantly compared to other topics. This increase is particularly evident in Topic-01, which was only a marginal topic in the AHP research about ten years ago. At present, however, this topic significantly dominates both in research interest and research impact. In terms of long-term development, the increase in the share in the AHP research can also be seen in Topic-04, although this increase was more significant about five years ago. The current trend suggests that the share of articles in Topic-01 will continue to grow significantly in the coming years and will likely dominate its research impact.

The following five topics can be considered as stable topics: Topic-03, Topic-05, Topic-06, Topic-08, and Topic-09. With these topics, it can be stated that their share is relatively stable over time, both in terms of research interest and research impact. The long-term trend suggests that there will probably be no significant changes in these topics, at least in the coming years.

We have identified two topics in which research impact and research interest are declining at the time—Topic-02 and Topic-07. Interestingly, these topics have dominated in the past and have been the main scientific currents in AHP research. Gradually, however, their importance was replaced by more current topics. In addition, Topic-02 was the most important topic in the past and is currently only an average melter. An even more marked decline can be observed at Topic-07—from the second most dominant topic to the least significant. Over the period of 30 years, its significance has been reduced three times. While Topic-02 can be expected to stabilize over time, the importance of Topic-07 is likely to continue to decline.

Based on the above results, it is possible to describe the main characteristics of the nine topics. We will focus mainly on their composition, representation in the subject area, their development over time, and at the same time, we will try to briefly list several studies that can be considered significant in the given topic with regard to the number of citations.

#### 3.3.1 Ecology & ecosystems (Topic 1)

In the first topic, there were mainly terms closely related to ecology and ecosystems. The most frequent terms in this topic were ’area’, ’water’, ’suitabl’, ’land’, ’region’ and ’potenti’. From the given composition of words, it can be concluded that the environmental focus of articles is dominant, which is directly related to ecology or ecosystem. Currently, this is a medium-sized topic (4441 articles), while from the point of view of the subject area, most articles are in the categories ENVI, EART, and AGRI. It follows that topic 1 is relatively clearly distinguishable from the others. At the same time, we can state that the development of this topic has recorded a significant growth only since 2005, not only in terms of research interest but also in terms of research impact. Our results suggest that this is the fastest-growing topic among all identified. We believe that this may be due to an intense increase in global climate problems, which is also reflected in scientific initiatives.

Several studies that have had a relatively good research impact can be pointed out in this topic. Pourghasemi, Pradhan and Gokceoglu [44] focused on the production of landslide susceptibility maps, comparing the results obtained through AHP and fuzzy logic. In an earlier study, Yalcin [45] also used AHP to create landslide susceptibility maps, using two other statistical index and weighting factor methods. The results showed slight deviations but were generally similar. Dai, Lee and Zhang [46] used AHP for geo-environmental evaluation for urban land-use planning. This is a relatively standard use of AHP as a decision-making tool that takes into account several criteria.

#### 3.3.2 Multi-criteria decision-making (Topic 2)

The second topic contained terms such as’ decis’, criteria ’,’ select ’,’ problem ’,’ altern ’and’ rank ’. It can be seen from the nature of these terms that they are closely related to decision-making, which is why we have called this topic multi-criteria decision-making (MCDM). This topic is the most extensive of all and contains 5041 articles. At the same time, the articles contain the most citations of all topics in the entire period. According to our findings, the research impact of AHP in multi-criteria decision-making is high. Articles focused on this topic fall mainly in the subject area ENGI, COMP, BUSI, DECI. If we look at the development of this topic, it was the dominant topic of the AHP until about 2008, while later other topics began to prevail. However, multi-criteria decision-making using AHP is still the second strongest topic in the last five years. Although the research impact of this topic is slightly declining in the long run, it is still one of the largest. This is one of the most important topics, and we believe that this is because it concerns the very essence of decision-making and its objectification. At the same time, AHP is not a tool in this topic but is directly an object of research.

Tzeng and Huang [47] pointed to the use of AHP in Multiple Attribute Decision Making (MADM). AHP in their book was one of the appropriate methods in addition to TOPSIS, VIKOR, ELECTRE, PROMETEE, fuzzy integrals, and rough set theory. Rezaei [48] published a relatively successful study, presenting a new decision-making method within MCDM and calling it BWM: best-worst method. Statistical results of this study show that BWM performs significantly better than AHP with respect to the consistency ratio and the other evaluation criteria: minimum violation, total deviation, and conformity. Two articles focused on the review of MCDM methods for sustainable energy decision-making also had a relatively significant impact on this topic [36, 49].

#### 3.3.3 Production and performance management (Topic 3)

Terms such as ’manag’, ’product’, ’perform’, ’industri’, ’implement’ and ’organ’ formed topic 3. The composition of these terms suggests that the topic is closely related to production and performance management. This is a relatively large topic (4781 articles) with a relatively high research impact measured over the total number of citations. Most articles on this topic have been published in the subject areas BUSI, ENGI, COMP, and DECI. The topic is really closely related to production and performance management. The number of articles dealing with these topic has become more significant since about 2000. Over the last 20 years, relatively stable usage of AHP in topics related to production and performance management can be seen—this applies to the number of articles as well as research impact. We believe that despite the growing objective side of decision-making in production and performance management, there are still types of decisions in which AHP finds application. At the same time, several studies with a significant impact on this topic can be mentioned.

Sarkis [32] used AHP to assess the green supply chain management elements and how they serve as a foundation for the decision framework. Handfield et al. [50] used AHP to evaluate the relative importance of various environmental traits and assess several suppliers’ relative performance along with these traits. Seuring [51] focused on sustainable supply chain management models, identifying AHP as one of the relevant approaches.

#### 3.3.4 Sustainable development (Topic 4)

In the fourth topic related to AHP, the most commonly used terms were ’develop’, ’sustain’, ’environment’, ’resource’, ’econom’ and ’environ’. The first term dominated, while the representation of others was additional information. Given the composition of these terms, the topic was named sustainable development. This is a smaller topic (3205 articles), while the total number of citations of these articles is also smaller. Most articles focused on sustainable development were from the subject areas ENVI, ENGI, SOCI, and AGRI. Until 2015, this was a relatively insignificant topic, but it has grown since 2016 and is currently one of the relevant areas with the use of AHP. As with the first topic, it can be deduced that the increase in articles focused on sustainable development and AHP is related to the increase in scientific interest in environmental topics.

Brandenburg et al. [52] literature review in which he focused on quantitative models for sustainable supply chain management. In this study, he analyzed 134 papers, identifying that AHP is one of the most widely used methods of SCM-related decision making. Wu and Webster [53] used AHP as part of a multi-criteria evaluation simulation of land development. The suitability of AHP as a tool for comprehensive Environmental Impact Assessment—EIA, for example, was analyzed by Ramanathan [40], who addressed its benefits and described its shortcomings.

#### 3.3.5 Computer networks, optimization and algorithms (Topic 5)

The fifth topic consisted mainly of terms such as ’design’, ’network’, ’optim’, ’base’, ’time’ and ’algorithm’. This topic was more heterogeneous in terms of meaning, so we chose its broader name—computer networks, optimization and algorithms. This is a medium-sized topic (4191 articles), and the total number of citations to these articles was small compared to other topics. Most articles on this topic have been published in the subject areas ENGI, COMP, MATH, and PHYS. Interest in this topic has been only marginal in the past, with a slight increase in the number of articles since about 2015. Studies published between 2002 and 2013 had the highest research impact. Compared to other topics, the ratio between research interest and research impact in this fifth topic is unfavorable—the number of articles is higher, but the number of citations is lower. This may be due to the fact that computer science or mathematics is an exact science and has more suitable tools such as AHP to solve scientific problems.

Song and Jamalipour [54] published a study in which AHP was used to decide the relative weights of evaluation criteria set according to user preferences and service applications as a base to rank the network alternatives. Lin et al. [55] focused on customer-driven product design, using AHP to evaluate the relative overall importance of customer requirements and design characteristics. Mouzon and Yildirim [56] used AHP to determine the ’best’ alternative among the solutions on the Pareto front.

#### 3.3.6 Service quality (Topic 6)

Articles in the sixth topic had terms such as ’qualiti’, ’servic’, ’import’, ’expert’, ’provid’ and ’inform’. Such terms are semantically most associated with the field of service quality, so we named the sixth topic this way. The sixth topic is medium in size (3670 articles), and its research impact is smaller (articles of this topic are not on average significantly cited compared to articles from another topic). Representation of service quality was in practically all subject areas, but the four most important are ENGI, SOCI, COMP, and MEDI. Topic service quality is the most stable topic in terms of time development—practically since 1994, it has been steadily equally represented and thus undoubtedly forms an important topic with history and current applications. The stability of the sixth topic was not only recorded in terms of the number of articles but also in terms of the number of citations. Given the global development of society and the transformation of many economies in terms of services, it is possible to see continuing interest in services and their quality. This could partly explain the above characteristics of this sixth topic.

The following three studies can be included among the most important studies. Cheever et al. [57] focused on prioritizing cancer antigens in a medical study and used AHP to deal with complex decisions. The second study is by Ho [13], which focused on the analysis of articles in which AHP is combined with other tools such as QFD, DEA, or SWOT, stating that integrating AHP with other methods is generally better than stand-alone AHP. A work by Forman and Gass [8] focused on exposing the reasons for AHP’s wide variety of applications and the efficacy and applicability of the AHP vis-a-vis competing methodologies.

#### 3.3.7 Fuzzy logic (Topic 7)

The seventh topic consisted of terms such as ’weight’, ’fuzzi’, ’valu’, ’determin’, ’obtain’ and ’base’. The first two terms dominated this topic, so we named it fuzzy logic. The size of the topic is smaller (2991 articles), but its research impact is higher compared to other topics. We believe that a small number of articles contain important information that is applicable to various subject areas. Articles focused on AHP and fuzzy logic were mainly from the subject areas ENGI, COMP, MATH and DECI. Given the time evolution of the topic, Fuzzy logic played a very important role in the AHP method, especially in the nineties. Research interest and research impact of fuzzy logic steadily declined. Nevertheless, it cannot be said that this is a dying topic. The "decline" of this topic is due to the faster growth of other topics, while fuzzy logic still finds significant applications in various subject areas.

One of the popular studies by Mikhailov [58] focused on deriving priorities from fuzzy pairwise comparison judgments is proposed, based on α-cuts decomposition of the fuzzy judgments into a series of interval comparisons. Six years later, Wang et al. [36] proposed extent analysis method on fuzzy AHP to obtain a crisp priority vector from a triangular fuzzy comparison matrix. They found that the extent analysis method cannot estimate the true weights from a fuzzy comparison matrix and has led to quite a number of misapplications in the literature. Another important study from Alonso and Lamata [59] presented a statistical criterion for accepting/rejecting the pairwise reciprocal comparison matrices in the analytic hierarchy process.

#### 3.3.8 Systematic evaluation (Topic 8)

We named the eighth topic systematic evaluation because it contained two dominant terms, ’evaluation’ and ’system’. In addition, other terms have been identified that can be considered complementary—’energy’, ’indic’, ’establish’ and ’power’. This is a medium-sized topic (3893 articles) with less research impact. More than a third of all articles (37%) on this topic were published in the subject area ENGI, followed by less represented areas such as ENER, COMP, and ENVI. This may be partly logical, as such links have already been shown to us in the previous analysis in Chapters 3.1 and 3.2. By the year 2000, this topic was practically negligible, but it gradually began to grow, and even in the period 2009–2014, it was one of the three most important. The growth of this topic around 2015 has stabilized and currently has approximately the same proportion of articles focused on AHP. Research interest exceeds research impact, which is comparable to less important topics. Nevertheless, there are several studies whose research impact has been relatively significant.

By far, the most significant research impact was recorded by an article by Thomas Saaty [19], author of AHP, who published a summary study on AHP. He presented principles and the philosophy of theory, and he summarized general background information of the type of measurement utilized, its properties, and applications. The impact of this study was enormous (4785 citations) and significantly affected a number of other subject areas. None of the other studies on this topic came close to the impact of Saaty’s work. This would partly explain the small research impact of the topic of systematic evaluation at present—we assume that current research refers to the original article published in 1990. The second study with much less impact—but not negligible, given the 383 citations—is by San Cristóbal [60]. In it, the author focuses on using AHP for weighting the importance of different criteria, which allows decision-makers to assign these values based on their preferences. Hermann, Kroeze and Jawjit [61] published a study in which they presented a new analytical tool, called COMPLIMENT, based on AHP, which can be used to provide detailed information on the overall environmental impact of a business.

#### 3.3.9 Risk assessment (Topic 9)

The last topic was formed by the terms ’risk’, ’assess’, ’project’, ’construct’, ’safeti’ and ’structur’. Since the first two terms dominated, we called this topic risk assessment. In terms of numbers, this topic is small (3218 articles) and has a correspondingly small research impact. The articles in this topic are from all research areas, but they are significantly dominated by the subject area ENGI (36%), followed by ENVI (7%), EART (7%), and COMP (7%). We assume that risk assessment dominates the most in ENGI, but it is relevant for practically all subject areas. The risk assessment topic has been relatively stable since about 2000 if we take into account the research interest. The research impact of this topic has been relatively stable since 2000. Studies with the highest impact can also be mentioned in this topic.

Esawi and Farag [62] used AHP to select the optimum material for a tennis racket. AHP was used in the decision-making phase, in which it was necessary to eliminate subjectivity and thus reduce the risk of a wrong decision. Yüksel and Daǧdeviren [63] also published a study using AHP in SWOT analysis. They state that although the AHP technique removes these deficiencies, it does not allow for the measurement of possible dependencies among the factors and therefore, they propose their own algorithm using ANP (analytical network process). The third major study was published by Kutlu and Ekmekçioǧlu [64] directly used the risk assessment tool—Failure mode and effects analysis (FMEA). In this study, a fuzzy approach was developed. It allows experts to use linguistic variables for determining S, O, and D, by applying fuzzy TOPSIS integrated with fuzzy AHP.

## 4 Discussion

### 4.1 Summary of main findings

We presented the main results of processing an extensive dataset of scientific documents in sections 3.1, 3.2 and 3.3. In the introduction to the article, we have defined three research questions, to which we will now try to find brief answers. At the same time, it should be noted that a more comprehensive answer to the questions can be found in Chapter 3.

RQ1: What is the usage of AHP and research impact in individual subject areas? Main highlights:

- ***The most represented areas of AHP use clearly include Engineering*** (ENGI 25.6%), followed by Computer Science (COMP 12.0%), Environmental Science (ENVI 10.3%), and Business, Management and Accounting (BUSI 8.4%).
- ***Publications on AHP achieve the highest research impact in the subject areas Decision Sciences*** (38.8 citations per article) ***and Mathematics*** (28.8 citations per article). This could be explained by the fact that these areas have been the basis for the development of AHP in the past, and so far, the authors deal with the very essence of AHP.

RQ2: What are the trends in the popularity of AHP from the first introduction of the method in 1980 to the present? Main highlights:

- ***The publication of AHP articles is growing very significantly over time***. In the last four years (2017–2021), more than 15,000 new articles with such a focus have been published.
- ***The highest increase in the total number of articles concerned the subject area Environmental Science***—by 2010, 582 articles had been published, and since 2010 it was 3,623 articles, which represents a more than 6-fold increase. For comparison, the most numerous subject area Engineering recorded an "only" 3.5-fold increase. With current trends, it can be expected that in 3 to 4 years, the subject area Environmental Science could already be the most numerous area in articles related to AHP.
- ***Interest in AHP has grown significantly among researchers in the subject area of Business*, *Management*, *and Accounting***—currently, one in 300 articles published in this field is focused on AHP (in 2005, it was 1 article in 1000 published). This topic has been consistently popular for almost two decades in Engineering (1 article in 250 published) and Decision Sciences (1 article in 300).

RQ3: What are the most common topics related to AHP, and what is their development over time? Main highlights:

- ***Nine fundamental topics related to AHP were identified*** by using machine learning methods: Ecology & Ecosystems; Multi-criteria decision-making; Production and performance management; Sustainable development; Computer network, optimization, and algorithms; Service quality; Fuzzy logic; Systematic evaluation; Risk assessment.
  ■ ***Ecology & Ecosystems***. A relatively new topic, probably related to the growing interest in environmental issues in the world; it has been growing significantly since about 2005.
  ■ ***Multi-criteria decision-making***. The most extensive topic in which the authors deal with the very essence and improvements of the AHP method.
  ■ ***Production and performance management***. A stable topic focused on the application of AHP to various aspects of managerial decision-making related to production and performance.
  ■ ***Sustainable development***. A topic with a rapid growth rate, which can be explained by the increasing intensity of sustainability research.
  ■ ***Computer network*, *optimization and algorithms***. A relatively heterogeneous topic that uses AHP to objectify decisions that other mathematical apparatuses cannot solve.
  ■ ***Service quality***. An extremely stable topic in which AHP is used in various aspects of service quality research.
  ■ ***Fuzzy logic***. The topic was dominant and showed a high research impact in the last century dealing with weights determination using fuzzy logic.
  ■ ***Systematic evaluation***. Practically oriented topic with less research impact focused on the use of AHP in technically oriented decisions, especially in the field of Engineering.
  ■ ***Risk assessment***. A smaller but stable topic covering themes related to the use of AHP in risk assessment in various application areas.
- ***The highest increase in the share in terms of time development was recorded by the topic Ecology & Ecosystems***. This applies to both research interest (number of articles) and research impact (number of citations).
- ***Articles related to AHP differ depending on the research object***. Multi-criteria decision-making and Fuzzy logic are two topics that deal with the very essence of AHP—principles, axioms, rules, and development—and AHP is directly the subject of their research. The other seven topics are used by AHP primarily as a tool for other various research objects.

A summary of the individual characteristics of the identified topics can be found in Table 2.

**Table 2:** Summary of topics characteristics.

Topic	Most related subject area	Research interest (number of articles)	Research impact (number of citations)	Top-3 cited articles	Longterm trend
Ecology & Ecosystems	ENVI	4441	53 463	[43, 44, 65]	Rising
Multi-criteria decision-making	ENGI	5041	114 254	[36, 46, 48]	Declining
Production and performance management	BUSI	4781	68 216	[32, 49, 50]	Stable
Sustainable development	ENVI	3205	38 173	[34, 51, 66]	Rising
Computer networks, optimization and algorithms	ENGI	4191	32 978	[67–69]	Stable
Service quality	ENGI	3670	41 844	[3, 70, 71]	Stable
Fuzzy logic	ENGI	2991	52 819	[7, 16, 72]	Declining
Systematic evaluation	ENGI	3893	27 252	[19, 34, 73]	Stable
Risk assessment	ENGI	3218	28 816	[60–62]	Stable

### 4.2 Theoretical and practical implications in production research

Our overview of the use of AHP offers a general picture of this universal method in different subject areas and different topics. If we take a closer look at studies that are directly focused on production research, we could identify three main areas.

The first area is the use of AHP in supply chain management. With regard to the composition of articles on SCM, it can be fairly argued that this is a top area with the use of AHP in the field of production research. This was confirmed, among other things, by an overview of the three most cited articles in the third topic, Production and Performance Management. The AHP is useful in SCM, especially if the research focuses on green SCM [74–78] or supplier evaluation or selection [33, 35, 79–85].

The second area is multi-criteria decision-making. Apart from the fact that this topic was also identified by our analysis, after a deeper examination of the articles in production research, we see a similar use. For example, Bhattacharya, Sarkar and Mukherjee [86] combined AHP with QFD to select a robot. This selection was based on requirement analysis, and AHP plays a role in weight determination requirements. Wei, Chien and Wang [87] also used AHP in 2005 to support ERP (Enterprise Resource Planning) selection decisions. In the later period, more advanced modifications of the AHP were used in production research decisions. Bouzon et al. [88] used fuzzy AHP to analyze reverse logistics barriers. Achieving optimal decision-making of cloud manufacturing service provided was the subject of a study by Hu et al. [89], while its authors used, in addition to AHP, other more advanced decision-making tools such as TOPSIS or Grey Correlation Analysis. AHP has also been used to support decision-making for logistics operations in distribution centers [90]. Last but not least, Ishizaka et al. [91] used AHP in conjunction with Data Envelopment Analysis (DEA) to multi-criteria inventory classification. It can be seen from this overview that AHP combines relatively well with other tools, whether they are requirements-based tools (such as QFD) or decision support tools (such as TOPSIS).

The third area in production research where AHP applications can be found is risk. We also identified this topic in our analysis. If we take a closer look at the articles focused on risk management or risk assessment, we can see a relatively wide range of applications. Samvedi, Jain and Chan [92] used fuzzy AHP and fuzzy TOPSIS to quantify risks in a supply chain. Dong and Cooper [93] pointed to the fact that the traditional assessment methodologies are unable to deal with intangible criteria, which are a crucial factor in the analysis. They developed an orders-of-magnitude AHP (OM-AHP) based ex-ante supply chain risk assessment model to compare the tangible and intangible elements that influence supply chain risks. Ilbahar et al. [94] used Pythagorean fuzzy AHP & fuzzy inference system to risk assessment for occupational health and safety, comparing the results with another risk assessment tool—FMEA (Failure Mode and Effect Analysis). Kumar et al. [95] use fuzzy AHP to prioritize the risks under vague and unclear surroundings.

In addition to these theoretical benefits, our research may have several practical implications. One of the most significant practical findings is that AHP is a truly universal decision-making tool, documented by more than 40 years of research. The use of AHP to objectify the work of decision-makers in the industry can have several levels—basic, advanced, and expert. At the basic level—for managers who do not have much experience with the systematic assessment of unstructured problems, the basic version of the AHP can already be a functional tool for qualitative-quantitative decision-making. The systematic evaluation was also one of the identified topics, while it was very widely represented almost in all subject areas—which testifies to the universality of AHP. At the advanced level—for managers who know and use simpler and moderately demanding decision support tools, AHP can help assess criteria through a multi-criteria decision-making process. In such cases, the AHP acts as a support tool, usually a multi-step decision-making tool. At the expert level—AHP can also be used in complex production systems to increase productivity, reduce risk or objectify strategic decisions. Solutions related to fuzzy logic can serve in such types of decisions, and even small improvements can bring significant economic and non-economic benefits in complex production systems.

### 4.3 Research limitations and future research opportunities

Several research limitations can also be identified in our research. Non-absolute indexing, which is the first limitation, refers to sampling bias due to the limitations of the Scopus database. The Scopus database does not index all scientific articles related to the AHP method. Some AHP-related articles may only be exclusive to the Web of Science or other databases. However, several studies suggest a significant overlap between the Scopus and Web of Science databases [96, 97]. At the same time, Scopus contains more than 76 million records, making it the world’s largest abstract and citation database. The sample of articles in this paper was very high (more than 34,000 documents), and from a statistical point of view, this can be considered as a representative and robust solution. At the same time, it should be noted that different databases (e.g., Scopus, Ebsco, Web of Science) have data in different structures—e.g., they have different subject areas. By combining databases, we would probably achieve a higher number of articles, but their data structure would not be consistent, and therefore we would not be able to answer the first two research questions objectively.

The second limitation is the partial inclusion of incorrect articles caused by synonymous terms. However, we assume that this proportion of articles was small enough and did not significantly affect the results of our research.

The third limitation is limited text analysis–we analyzed only abstracts of articles. On the other hand, we assume that this shortcoming was not major because the abstract usually contains the most relevant information. The median number of processed words in the abstract was 185.

The fourth limitation is the absence of a complete PRISMA methodology used for articles such as systematic literature reviews. It should be noted that our article is not a standard type of systematic literature review article. Our article does not focus on in-depth analysis of a limited number of relevant articles but uses big data approaches and machine learning tools to cover the topic of AHP as comprehensively as possible. It is important to emphasize that study is not a standard type of systematic literature review. We do not focus on a specific issue in a specific area of interest—we focus on a comprehensive overview of AHP in all areas. Such approaches to literature reviews are currently beginning to be applied to a number of rapidly evolving topics [98, 99].

Our approach offers also several opportunities for further research. First of all, a multistage analysis of topics can be mentioned. We analyzed the entire dataset of documents when identifying topics, which helped us identify the most prominent "macro" topics. Each topic could be subjected to further analysis to identify more detailed "micro" topics. This could contribute to a better understanding of the development of AHP and its use in scientific work.

Another potential is the application suitability of LDA and the robustness of its results. According to current data from the Scopus database, more than 5,000 documents concern Latent Dirichlet Allocation, of which approximately 11% also contain the keywords “review”. In most cases, LDA was used to model topical practice-oriented topics. The use of LDA for the analysis of topics in online reviews was used by Guo, Barnes and Jia [100] or Tirunillai and Tellis [101]. Calheiros, Moro and Rita [102] used LDA to gather relevant topics that characterize a given hospitality issue by a sentiment. Boussalis and Coan [103] focused on analyzing the signals of climate change doubt, using the LDA on more than 16,000 documents from 19 organizations between 1998 and 2013.

The LDA has only been used in recent years to analyze topics in research areas. D’Amato et al. [104] used bibliometric data to analyze the green, circular, and bioeconomy areas. Mäntylä, Graziotin and Kuutila [105] used the evolution of sentiment analysis to analyze nearly 7,000 documents from the Scopus database. According to some sources, unstructured data (eg text) represents more than 80% of all data [106]. Thus, LDA appears to be a suitable method for research on topics, enabling it to cover a large number of documents and extract relatively meaningful and interpretable results. In this article, we have focused on the analysis of the use of the AHP method, but LDA can be applied to virtually any research theme in which the analysis of topics is relevant. We assume that the use of LDA for topic analysis in various research areas will grow.

## 5 Conclusion

There are a large number of AHP applications, and it is quite difficult to capture them in all their complexity. Although standard literature reviews offer an up-to-date view of the most important publications, they naturally cannot cover the topic in its entirety. Our approach to literature review-based LDA topic modeling is the first to be used on the AHP. This probabilistic clustering approach makes it possible to process a large amount of information and identify the most frequent topics in the corpus of documents.

In our study, we analyzed more than 35,000 abstracts of scientific documents from the Scopus database related to AHP. We cover three research questions with our analysis. The first was to identify the usage of AHP and its research impact in individual subject areas. In the second research question, we focused on the analysis of trends in the popularity of AHP from the first introduction of the method in 1980 to the present. We analyzed the trends with regard to individual subject areas. The third research question was to capture the time evolution of AHP-related topics. We identified nine topics, which we subjected to a deeper statistical survey, and we captured the development over time with regard to the research interest and research impact of each topic.

Given the long-term growth trend of articles focused on or using AHP, our results can offer an up-to-date and robust information base for further research. We believe that our study can provide a basis for a broader scientific discussion on AHP. We also believe that the use of topic modeling has great potential in the literature review in any research area.
